# Spatial Colinear but Broken Temporal Expression of Duplicated *ParaHox* Genes in Asexually Reproducing Annelids, *Nais communis* and *Pristina longiseta*

**DOI:** 10.3390/genes14071501

**Published:** 2023-07-22

**Authors:** Roman P. Kostyuchenko, Artem V. Amosov

**Affiliations:** Department of Embryology, St. Petersburg State University, Universitetskaya nab. 7-9, 199034 St. Petersburg, Russia; artem221199@mail.ru

**Keywords:** ParaHox, gene duplication, asexual reproduction, nervous system, posterior growth zone, gut regionalization, tissue remodeling, axial patterning, Annelida, evolution

## Abstract

ParaHox genes are key developmental regulators involved in the patterning of the digestive tract along the anteroposterior axis and the development of the nervous system. Most studies have focused on the function of these genes in embryogenesis, while their expression patterns in postembryonic development often remain unknown. In this study, we identified for the first time all *ParaHox* orthologs in two naidid oligochaetes, *N. communis* and *P. longiseta*, and described their expression patterns during normal growth and fission in these animals. We showed that *Gsx* and *Cdx* are presented by two paralogs, while *Xlox* is a single copy gene in both species. Using whole-mount in situ hybridization, we also found that orthologs, except for the *Xlox* gene, have similar activity patterns with minor differences in details, while the expression patterns of paralogs can differ significantly. However, all these genes are involved in axial patterning and/or in tissue remodeling during growth and asexual reproduction in naidids. Moreover, during paratomic fission, these genes are expressed with spatial colinearity but temporal colinearity is broken. The results of this study may be evidence of the functional diversification of duplicated genes and suggest involvement of the ParaHox genes in whole-body patterning during growth and asexual reproduction in annelids.

## 1. Introduction

Transcription factors that encode ParaHox genes play crucial roles in controlling development and specification of different organs and tissues in bilaterian animals. ParaHox and Hox genes are believed to be evolutionary sister groups evolved from an ancient Proto-Hox/ParaHox cluster prior to the divergence of cnidarians and bilaterians [1,2,3,4]. Nevertheless, functional diversification of these genes has been proposed. Thus, it has been hypothesized that the ancestral role of Hox genes was primarily in anterior–posterior patterning and specification of ectodermal and neuroectodermal tissues and organs during development. In contrast, ParaHox genes might play a key role in anterior–posterior patterning in the digestive tract anlagen, especially endodermal [2,5]. Thus, three ParaHox genes (*Gsx*, *Xlox*, and *Cdx*) originating from the Proto-Hox/ParaHox cluster are thought to have been involved in patterning the anterior, middle, and posterior gut regions in a colinear manner in basal animals [1,5].

ParaHox genes were first described as a gene family in 1998 in the amphioxus [1]. A number of works describing their expression in model and non-model organisms support the idea of an ancestral role of these genes in the patterning of the digestive tract. On the other hand, many studies also showed that each of the three ParaHox genes could be involved in patterning, not only the gut, but also the nervous system of the bilaterian ancestor [6,7].

The cluster organization of ANTP-class genes (Hox, ParaHox, and NK) is believed to be important in the evolution and functioning of these genes, including the regulation of temporal colinear expression. The spatial colinearity of expression can be realized even in the absence of intact clusters [2,3,4]. Many deuterostomes, with the exception of the sea urchin, ascidians, and some bony fishes [8,9,10], are characterized by the presence of intact clusters with the same transcriptional orientation and gene sequence *Gsx-Xlox-Cdx* [1,3,11,12]. In addition to full and duplicated clusters, dispersed clusters, separate paralogs, and even gene loss were also often described in different deuterostomes [10].

In contrast, protostomes show a greater diversity in the organization of ParaHox genes. Ecdysozoans are characterized by cluster disintegration, as well as gene loss. No *Xlox* homologs have been found in any of the studied animals from this clade. *Gsx* is also absent in nematodes. Lophotrochozoans, except for flatworms, are characterized by the presence of all three genes, but their location in the genomes varies [6,13].

Data on the expression of *Gsx*, unlike the other two ParaHox genes, cause the most discussion in the context of its ancestral function in the patterning of the anterior digestive tract. In deuterostomes, expression of this gene is shown in anterior neural structures [1,8,14]. Among lophotrochozoans, in nereidid annelids, *Gsx* expression is described in the neural anlagen, as well as in the stomodeum at the trochophore stage and later in the foregut and midgut [6,15]. In contrast, *Capitella teleta* only shows neural expression during development [16]. A diversity of patterns is also observed among molluscs. In Scaphopoda and Cephalopoda, *Gsx* is expressed only in neural structures [7]; in gastropod *Gibbula varia*, both neural and stomodeal expression are shown [17].

Data on *Xlox* (*Pdx* or *Ipf* in deuterostomes) expression are limited but mainly support the ancestral role of this gene in patterning of the gut and, possibly, the nervous system. In vertebrates, it is expressed in both the gut [18,19] and the nervous system anlagen [20]. In addition, endodermal and neural expression of *Xlox* was also shown in amphioxus and starfish larvae [1,11].

In two nereidid annelids, *Platynereis dumereilii* and *Alitta virens*, both neural and endodermal expression of *Xlox* is described [6,15]. For other annelids such as *C. teleta* and the leeches *Helobdella robusta* and *Hirudo medicinalis*, only endodermal *Xlox* expression is known [16,21]. Additionally, in the developing mollusc *G. varia,* this gene expression suggests its ancestral role in the gut and nervous system [17].

The most “posterior” ParaHox gene, *Cdx* or *caudal*, is characterized by the greatest diversity of expression patterns, both spatial and temporal, as well as a variety of known functions. Unlike the first two genes, *Cdx* is known to be expressed in all three germ layers. In deuterostomes, orthologs of this gene are expressed in the hindgut. In mammals, specification of intestinal morphology by one of the three paralogs, *Cdx2*, has been proven by mutagenesis [22]. Moreover, *Cdx2* plays a critical role in blastomere differentiation into trophectoderm [23]. Functions of *Cdx* are not limited to the formation of the morphology of the intestinal epithelium. *Cdx* homologs play a role in the patterning of the anterior–posterior axis through the regulation of colinear expression of Hox genes in the paraxial mesoderm [24] and in elongation of the main body axis. The latter may be ancestral to all bilaterians [25,26].

In both arthropods and nematodes, *Cdx* expression is showed in all germ layers in the posterior part of the body [25,27,28].

Lophotrochozoans, except for the oligochaete *Tubifex tubifex* and the gastropod *Patella vulgata*, show *Cdx* expression in the endo- and ectodermal domains of the hindgut. In the oligochaete *T. tubifex*, expression is described in the blast cells, the descendants of mesodermal teloblasts [29]. In the gastropod *P. vulgata*, it is also shown in mesodermal teloblasts formed from the 4d blastomere at the trochophore stage, but also in the prototroch and later in the mesoderm and neuroectoderm on the ventral side [30]. Apparently, the absence of expression in the hindgut in *P. vulgata* can be considered as secondary, since in other studied gastropods (*Tritia obsoleta*, *G. varia*, and *Crepidula fornicata*) such expression is present, and the hindgut is also formed from descendant cells of the 4d blastomere [17,31,32].

Among annelids, nereidids show similar patterns of *Cdx* expression in the posterior part of the blastopore and later in the hindgut and pygidium [6,15]. *C. teleta* displays unusual *Cdx* expression in all germ layers, including the anterior anlagen, that has not been reported for other annelids [16].

Despite a significant amount of data, our knowledge of these genes remains incomplete, since most studies concern the function of these genes in embryogenesis, while their expression patterns in postembryonic development often remain unknown.

The freshwater naidid annelids (Clitellata, Naididae), *Nais communis* and *Pristina longiseta*, are excellent models to investigate different forms of postembryonic development such as growth, asexual reproduction, and regeneration [33,34,35,36,37,38,39]. They are tiny and mostly transparent. In long-term laboratory culture both species reproduce only by paratomy, a type of agametic propagation in which new head and tail ends develop within one of the middle body segments before the parent organism physically separates into daughter individuals. As a result, the animals form temporary chains of zooids that break away from each other when the new structures are completely formed. The paratomic fission of naidids is well morphologically described [34,35,38]. It is characterized by active cell proliferation, formation of blastema masses of undifferentiated (possibly dedifferentiated) cells, and remodeling of old tissues. At the initial events, the paratomy zone is morphologically indistinguishable, but very soon it forms two regions with a distinct border. The anterior part of the fission zone, called somatogenic, develops a new posterior end of the anterior zooid. The posterior part (cephalogenic) of the fission zones gives rise to a new head end (including head segments, four in *Nais* and six in *Pristina*) of the posterior zooid [34].

Despite the similarity of the main events, the asexual reproduction of *N. communis* and *P. longiseta* has significant differences. *N. communis* forms temporary chains consisting of no more than two zooids, while *P. longiseta*, under favorable cultivation conditions, can develop multiple fission zones, and every additional fission zone is usually initiated in progressively more anterior segments. In contrast to the *slow paratomy* in *Nais*, in *Pristina*, fission is called *rapid paratomy*. Moreover, at the end of the asexual reproduction, *Pristina* shows remodeling of the gut of the original segments behind the new head into the new stomach by morphallaxis. Thus, at a certain level along the anterior–posterior axis, not only do new structures develop, but also a change in the identity of the segments occurs [34,35,40]. In contrast, *N. communis* does not have a morphologically distinct stomach and no remodeling of old gut tissues is observed. Thus, the process of patterning of new individuals occurs within the body of the parent, which requires a change in the expression of key developmental genes along the anterior–posterior axis. For example, in *P. longiseta*, the *Plo-otx* and *Plo-six3* genes show early expression in the segment in which the fission zone will develop, and thus mark the anterior border of the posterior zooid at early stages [35,41]. Despite the obvious advantages of this model for studying the (re)patterning processes during postembryonic development, our knowledge about the molecular mechanisms of body remodeling in adult bilaterians, in particular annelids, is very limited [35,41,42,43,44,45].

In this study, we cloned orthologs of all three ParaHox genes, *Gsx*, *Xlox*, and *Cdx,* for both *N. communis* and *P. longiseta* and examined their expression patterns by whole-mount in situ hybridization during growth and asexual reproduction in adult animals. We showed two duplications of ParaHox genes in studied species that support the data on the expansion of homeobox-containing genes in clitellates. We also found that during paratomic fission, these genes are expressed with spatial colinearity but temporal colinearity is broken. Our results suggest involvement of ParaHox genes in body patterning during asexual reproduction and growth in naidid annelids and may indicate functional diversification of duplicated genes.

## 2. Materials and Methods

### 2.1. Animal Material and Fixation

The specimens, originally found in a pond in the park of Biological Institute of Saint-Petersburg State University (St. Petersburg, Russia) in 1999, were used to maintain the laboratory cultures of both species, *Pristina longiseta* and *Nais communis*. According to [34], animals were cultured at 18℃ in Petri dishes with artificial spring water and Chlorophyta algae. An artificial illumination (16 h day, 8 h night) was used to optimize intensity of asexual reproduction in cultures. Mashed spinach or dried spirulina powder was used as feed.

To collect the material for in situ hybridization, worms from the main laboratory culture of each species were placed in separate Petri dishes with clean water in order to synchronize the animals. A week later, the worms were fed to stimulate asexual reproduction. From the next day, they were fixed sequentially every day, thus obtaining material from one to six days after stimulation. These samples were used to study the expression at the early stages of paratomy, when a morphologically distinct fission zone does not yet appear. Separately, worms with the developing fission zone as well as growing adults were fixed. Before fixation, the animals were anesthetized in relaxant solution for 3 min (10 mM MgCl_2_, 5 mM NaCl, 1 mM KCl, 8% ethanol [38]). The specimens were fixed in 4% PFA in PBS with 0.1% Tween 20 at +4 °C overnight. The fixed material was stored in MeOH at −20 °C.

### 2.2. Sequence Retrieval and Phylogenetic Analysis

For *Nais communis*, the sequences of *ParaHox* genes were identified in an unannotated transcriptome database (local resource). For *Pristina longiseta*, the *ParaHox* gene fragments were obtained first by degenerate PCR and then by RACE PCR. Amino acid alignment of the homeodomains was performed with MAFFT [46] using the Unipro UGENE v47.0 software [47]. Domain organization of the sequences was established using the online program PROSITE (https://prosite.expasy.org/, accessed on 15 December 2021). Bayesian phylogenetic analysis was conducted using the Markov chain Monte Carlo method implemented in MrBayes 3.2.7 (https://www.phylo.org/, accessed on 08 July 2023) [48]. The number of substitution types was fixed to 6. The search for best substitution model was performed in MEGA software v11.0.13 [49] and the LG model was chosen, while rate variation across sites was fixed to “invgamma”. Four Markov Chain Monte Carlo (MCMC) chains were run for 100,000 generations, sampling every 100 generations, with the first 250 sampled trees discarded as “burn-in”. Finally, a 50% majority rule consensus tree was constructed. The phylogenetic tree was handled using the FigTree program, v1.4.4 (http://tree.bio.ed.ac.uk/software/ (accessed on 08 July 2023).

### 2.3. Gene Cloning

In order to search *P. longiseta ParaHox* genes, degenerate PCR with asexual reproducing worms’ cDNA was performed. All primers are given in the Supplementary Materials (Table S1). The extended gene fragments were amplified by 5′-RACE and 3′-RACE PCR with gene-specific primers and cDNA prepared with SMARTer RACE cDNA Amplification Kit (Clontech, Cat. #634923). To validate overlapping of the *P. longiseta* 5′-RACE and 3′-RACE gene fragments inside the homeobox, as well as to isolate the *N. communis ParaHox* gene fragments, PCRs with gene-specific primers and cDNAs were carried out. These amplified gene fragments were cloned and used for RNA probe synthesis. The PCR products were inserted in pCRII vectors by using TOPO-TA cloning kit (Invitrogen, Cat. #K4600-01) and used in the transformation of chemically competent *E. coli* (One Shot^®^ TOP10; Invitrogen, Cat. #K4600-01). The obtained colonies with the correct insert were checked by sequencing. As a result, except for *Nco-Gsx2* and *Plo-Gsx2*, all amplified fragments include complete CDS, 5′ and 3′UTR. Sequences of *Nco-Gsx1* (1440 bp), *Nco-Gsx2* (1223 bp), *Nco-Xlox* (1834 bp), *Nco-Cdx1* (2100 bp), *Nco-Cdx2* (1746 bp), *Plo-Gsx1* (1074 bp), *Plo-Gsx2* (1061 bp), *Plo-Xlox* (1089 bp), *Plo-Cdx1* (1243 bp), and *Plo-Cdx2* (1667 bp) are deposited in GenBank with the accession numbers OR050790-OR050799. A 5′ partial CDS of *Plo-Cdx2* cDNA (1507 bp) was cloned previously (GenBank JQ685130).

### 2.4. Whole-Mount In Situ Hybridization

The whole-mount in situ hybridization (WMISH) experiments were performed as previously described [50,51] with several modifications. Templates for the *Nco-Gsx1*, *Nco-Gsx2*, *Nco-Xlox*, *Nco-Cdx1*, *Nco-Cdx2*, *Plo-Gsx1*, *Plo-Gsx2*, *Plo-Xlox*, *Plo-Cdx1*, and *Plo-Cdx2* digoxigenin labeled RNA probes (antisense and sense) were, respectively, ~1250 bp (positions 96–1349 in GenBank OR050790), ~1189 bp (positions 1–1189 in GenBank OR050791), ~1300 bp (positions 211–1512 in GenBank OR050792), ~1330 bp (positions 75–1401 in GenBank OR050793), ~1160 bp (positions 221–1384 in GenBank OR050794), ~760 bp (positions 89–851 in GenBank OR050795), ~870 bp (positions 66–934 in GenBank OR050796), ~850 bp (positions 102–955 in GenBank OR050797), ~1025 bp (positions 54–1079 in GenBank OR050798), and 1507 bp (positions 1–1507 in GenBank OR050799). A previously cloned fragment of *Plo-pax6* gene (GenBank OR258068), that encodes a transcription factor involved in the development of the central nervous system, was used as a template for the digoxigenin labeled RNA probe ~1200 bp in length. After rehydration, fixed material was treated with proteinase K (100 mkg/ml; Merck, Cat. #1.24568.0100) for 2 min at +22 °C and stopped by washing twice in glycine in PTw (2 mg/mL). Then the objects were postfixed with 4% PFA in PTw for 20 min. Prior to the pre-hybridization step, the samples were washed several times in PTw. After incubation with the DIG-probe at +65 °C, subsequent washes, incubation with anti-digoxigenin AP antibodies (1:2500; Roche, Cat. #1093274910), and washing, the objects were stained with NBT/BCIP (Roche, Cat. #11383213001/11383221001). After washing in PTw, specimens were mounted in 90% glycerol. In situ hybridization with the sense DIG-labeled riboprobe was used as a negative control.

### 2.5. Immunohistochemistry

The growing adults and asexual reproducing *P. longiseta* worms were first anesthetized and then fixed as described above (Section 2.1. Animal material and fixation). All further steps were carried out at room temperature, unless stated otherwise. After several rinses in PBS with 0.1% Tween 20 and permeabilization with 0.1% Triton-X in PBS (PBT), specimens were preincubated in 5% normal seep serum (Sigma, Kawasaki city, Japan, Cat. #S2263) in PBT for 1 h. Following preincubation, the materials were incubated overnight at 4 °C in PBT with primary antibodies against FMRF-amide (polyclonal rabbit anti-FMRF-amide in a dilution of 1:400; ImmunoStar, Hudson, WI, USA, Cat. #20091). Specimens were then washed in PBT several times and incubated in PBT with Alexa-Fluor-488-conjugated goat anti-rabbit IgG (H + L) antibodies (1:400; Invitrogen, Waltham, MA, USA, Cat. #A-11008) and DAPI (1 mkg/mL) for 2 h. After washing with PBT and PBS, specimens were mounted in 90% glycerol and examined by confocal laser scanning microscopy.

### 2.6. Data Visualization

Results of in situ hybridization were visualized using DIC optics with an Axio Imager D1 microscope (Carl Zeiss, Oberkochen, Germany). Digital photomicrographs were taken with an AxioCam ICc3 digital camera using AxioVision 4.8 software (Carl Zeiss, Germany).

After immunohistochemistry, the mounted specimens were imaged using a Leica SP5X confocal laser scanning microscope (Leica, Wetzlar, Germany). Z-stacks with 1.0 mkm steps were acquired using the Leica LAS X Office software.

The artworks were made in ImageJ, MS PowerPoint (Microsoft Office 2013), and Inkscape 1.2.2.

## 3. Results

### 3.1. Sequence Analysis

**Figure 1:**
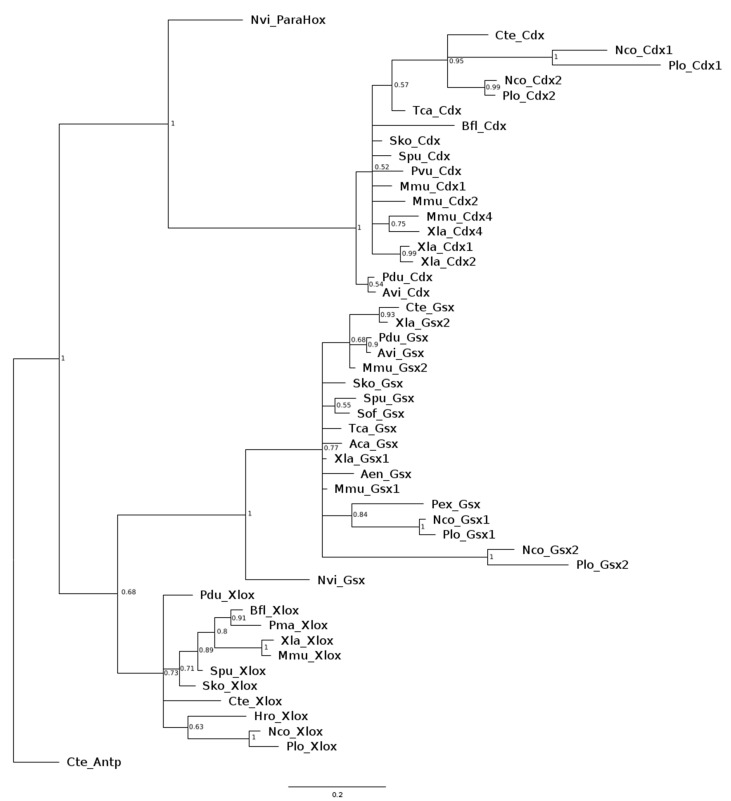
Phylogenetic analysis of *Nais communis* and *Pristina longiseta ParaHox* genes. Bayesian consensus tree of the homeodomains of metazoan ParaHox genes. The sequence of *Antp Hox* gene from *Capitella teleta* was used as an outgroup. Multiple alignment of ParaHox sequences used for phylogenetic analysis, see Supplementary Materials (Figure S1); GenBank accession numbers for sequences used for Gsx, Xlox, and Cdx amino acid alignments, see also Supplementary Materials (Table S2). Numbers at the nodes indicate posterior probabilities. Nco—*Nais communis*, Plo—*Pristina longiseta*, Aca—*Aplysia californica*, Aen—*Antalis entails*, Avi—*Alitta virens*, Bfl—*Branchiostoma floridae*, Cte—*Capitella teleta*, Ema—*Elysia marginata*, Hro—*Helodbella robusta*, Mmu—*Mus musculus*, Nvi—*Nematostella vectensis*, Pex—*Perionyx excavates*, Pdu—*Platynereis dumerilii*, Pma—*Pecten maximus*, Pvu—*Patella vulgate*, Sko—*Saccoglossus kowalevskii*, Sof—*Sepia officinalis*, Spu—*Strongylocentrotus purpuratus*, Tca—*Tribolium castaneum*, Xla—*Xenopus laevis*.

A phylogenetic tree constructed using the Bayesian Inference method demonstrates that cloned homologs from both *Nais communis* and *Pristina longiseta* are clustered with sequences obtained from the NCBI database into three clades corresponding to three ParaHox genes—*Gsx*, *Xlox*, and *Cdx*, where *Cdx* is a sister group to *Xlox* and *Gsx* (Figure 1). In most of the cases, studied genes are presented in the clades, containing homologous genes from closely related species and usually other members of the Clitellata group: *Cdx* genes with ortholog from *C. teleta*, *Gsx1* orthologs with *Gsx* gene from *Peryonix excavates,* and *Xlox* with an ortholog from *H. robusta.* This clustering confirms the success of the molecular cloning procedure. Thus, we identified in *N. communis* two paralogs of *Gsx, Nco-Gsx1* (GenBank OR050790) and *Nco-Gsx2* (GenBank OR050791), two paralogs of *Cdx, Nco-Cdx1* (GenBank OR050793) and *Nco-Cdx2* (GenBank OR050794), and one ortholog of *Xlox*, *Nco-Xlox* (GenBank OR050792). For *ParaHox* genes in *N. communis*, we obtained, respectively, 1440 bp, 1223 bp, 2100 bp, 1746 bp, and 1834 bp of sequences. Similarly, for *P. longiseta*, we identified two paralogs of *Gsx, Plo-Gsx1* (GenBank OR050795) and *Plo-Gsx2* (GenBank OR050796), two paralogs of *Cdx, Plo-Cdx1* (GenBank OR050798) and *Plo-Cdx2* (GenBank OR050799), and one ortholog of *Xlox*, *Plo-Xlox* (GenBank OR050797). For *P. longiseta,* the sequences were, respectively, 1074 bp, 1061 bp, 1243 bp, 1667 bp, and 1089 bp in length. For both species, all sequences included 5′UTR and full homeobox, although *Gsx2* ortholog sequences, *Nco-Gsx2* and *Plo-Gsx2,* contained 3′-partial CDS.

The relationships between *Gsx* paralogs from the considered species remains unclear due to low support values leading to polytomy. However, there are specific sister clades containing *Gsx1* and *Gsx2* orthologs from *N.communis* and *P.longiseta* with high posterior probability, indicating duplication of these genes that happened before the diversification of these species.

*Cdx* orthologs also form a Naididae-specific clade. Based on this clade and the results of expression analysis of *Cdx2* in studied species, we can propose the hypothesis of *Cdx* evolution in the ancestors of the Naididae family, where the duplication of this *ParaHox* gene led to the diversification of its functions and expression patterns. However, it is worth noting that there is a lack of information in the databases regarding animals from the Lophotrochozoa group known to have ParaHox gene duplications. For this reason, the time when gene duplications, discovered in our study, have happened, is difficult to define.

Despite this, our analysis shows that there are two duplications of ParaHox genes in the studied Naididae species. *Gsx* and *Cdx* are presented by two paralogs, while *Xlox* is a single copy gene in both *N. communis* and *P. longiseta.*

### 3.2. ParaHox Gene Expression in Growing Adult N. communis and P. longiseta

ParaHox gene expression was examined throughout asexual reproduction and growing stages of *N. communis* and *P. longiseta* by WMISH. Orthologs, with the exception of *Xlox* genes, show similar activity patterns with minor differences in details.

**Figure 2:**
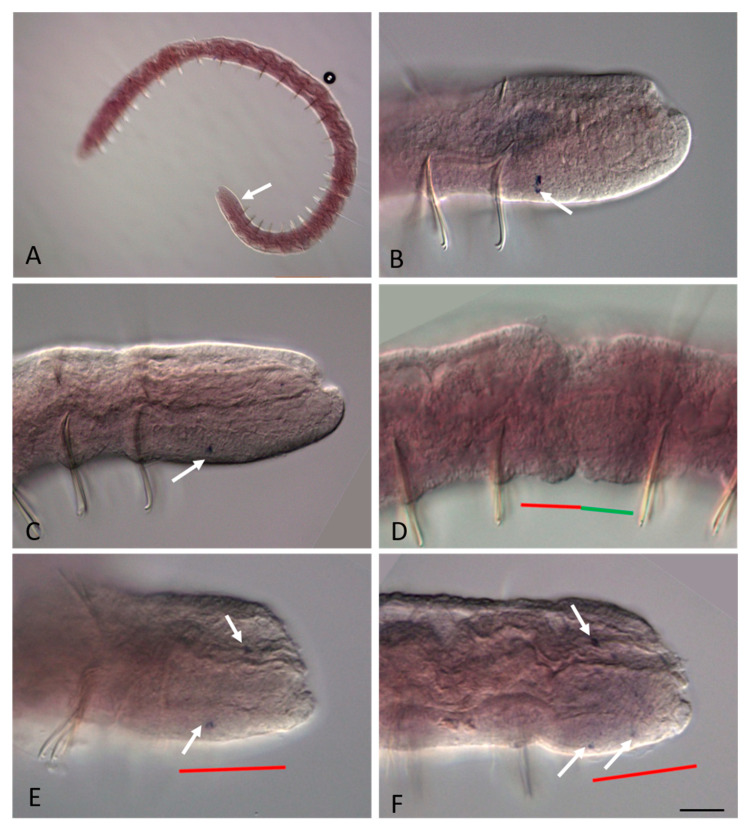
*Nco-Gsx1* expression during the growth and asexual reproduction by paratomic fission in *Nais communis*. All animals are oriented with the anterior to the left. (**A**) *Nco-Gsx1* is weakly expressed in a few cells (arrow) on the ventral side of the newly developed segments of a growing *N. communis* worm, lateral view. (**B**) Enlarged view of the posterior terminal region of the animal shown in (**A**). (**C**) Enlarged view of the caudal region of an asexual reproducing worm, early stage of paratomy. (**D**) No expression was detected in the cells of the fission zone at the middle stage of the paratomy, lateral view. (**E**,**F**) *Nco-Gsx1* signal becomes visible in single cells (arrow) on the ventral side of the newly developed segments in the anterior zooid at the late stage of paratomy. The posterior zooid was removed during in situ hybridization procedures. The red line marks the new developing tail region, and the green line marks the new developing head region within the fission zone. Scale bar, 30 mkm for all panels except (**A**). Scale bar in (**A**), 70 mkm.

**Figure 3:**
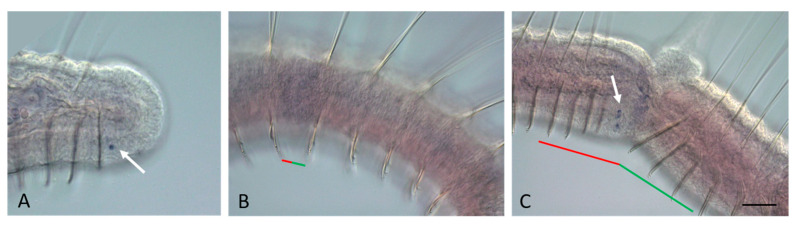
*Plo-Gsx1* expression during the growth and asexual reproduction by paratomic fission in *Pristina longiseta*. All animals are oriented with the anterior to the left, lateral view. (**A**) *Plo-Gsx1* is expressed in a few cells (arrow) on the ventral side of the newly developed segments of a growing worm. (**B**) No clear expression was detected in the cells of the fission zone at the early-middle stage of the paratomy. (**C**) *Plo-Gsx1* transcripts are detected in single cells (arrow) on the ventral side of the newly developed segments in the anterior zooid at the late stage of paratomy. The red line marks the new developing tail region, and the green line marks the newly developing head region within the fission zone. Scale bar in (**A**), 35 mkm. Scale bar in (**B**,**C**), 45 mkm.

During the growth stage of animals, both *Nco-Gsx1* and *Plo-Gsx1* are expressed at a very low level in a few cells on the ventral side in young segments that do not have formed setae (Figure 2A,B and Figure 3A). The transcripts are detected in the surface cells of the new segments; they are also found in a few cells inside the body.

**Figure 4:**
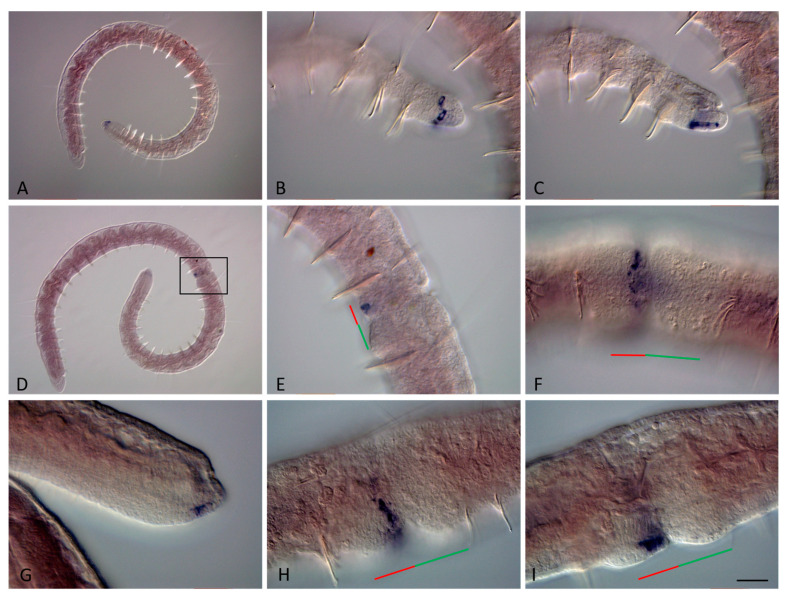
*Nco-Gsx2* expression during the growth and asexual reproduction by paratomic fission in *Nais communis*. Except for (**E**), all animals are oriented with the anterior to the left, in (**E**) the anterior is up. Lateral view for all panels. (**A**) *Nco-Gsx2* is strongly expressed on the lateral and ventral sides of the newly developed segments of a growing *N. communis* worm. (**B**,**C**) Enlarged view of the posterior terminal region of the animal shown in (**A**), different focal planes. (**D**) Within the middle-staged fission zone, expression is detected in the cells of the new developing tail region, where a new posterior growth zone is established. (**E**) Enlarged view of the boxed region shown in (**D**), deep focal plane. (**F**) *Nco-Gsx2* signal in the superficial cells on the lateral sides of the new developing tail region of the anterior zooid, middle stage of fission. (**G**) Caudal terminal region of a specimen at the middle paratomy stage. (**H**,**I**) Strong expression is observed in the cells of the new posterior growth zone and the developing, new structure of the ventral nerve cord at the late stage of paratomic fission, different focal planes. The red line marks the new developing tail region, and the green line marks the new developing head region within the fission zone. Scale bar, 45 mkm for all panels except (**A**,**D**). Scale bar in (**A**,**D**), 70 mkm.

**Figure 5:**
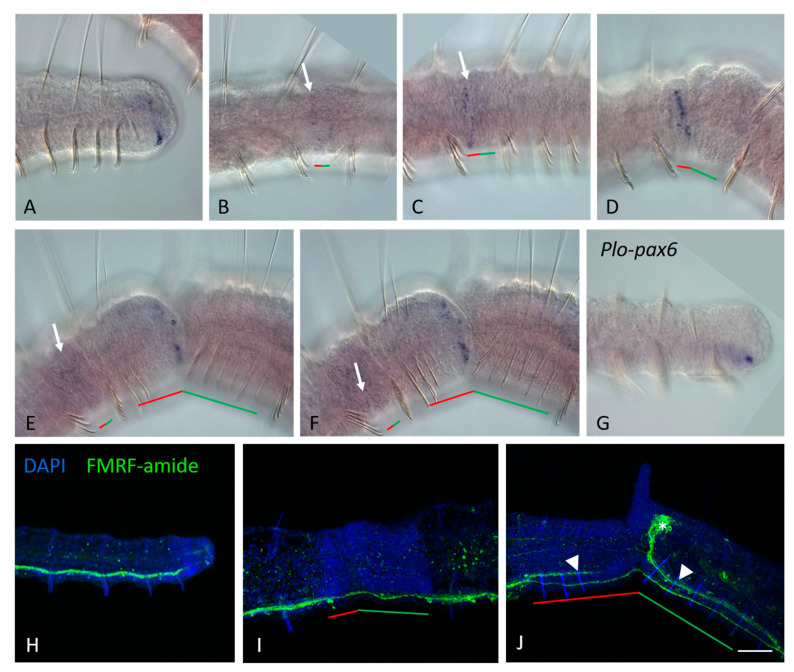
*Plo-Gsx2* (**A**–**F**) and neural marker (**G**–**J**) expression during the growth and asexual reproduction by paratomic fission in *Pristina longiseta*. All animals are oriented with the anterior to the left, lateral view. (**A**) *Plo-Gsx2* is expressed on the lateral and ventral sides of the newly developed segments of a growing *P. longiseta* worm. (**B**) A weak diffuse expression of *Plo-Gsx2* is observed in superficial cells (arrow) on the lateral sides of the new developing tail region of the anterior zooid, early stage of fission. (**C**) *Plo-Gsx2* expression becomes stronger (arrow) at the early-middle stage of paratomy. (**D**) Within the middle stage fission zone, *Plo-Gsx2* transcripts are detected in the cells in the new developing tail region, where a new posterior growth zone is established. (**E**,**F**) *Plo-Gsx2* expression is observed in the cells of the new posterior growth zone and the developing, new structure of the ventral nerve cord at the late stage of paratomic fission, different focal planes. At the same panels, a weak diffuse expression is observed in superficial cells (arrow) in an additional fission zone. (**G**) Plo-pax6, a marker gene of the developing central nervous system, is expressed on the ventral side of the tail end in a growing animal. (**H**–**J**) The nervous system in the growing and asexual reproducing P. longiseta as shown from immunoreactivity against FMRF-amide. Maximum projections of confocal Z-stacks scanned from lateral side. FMRF-amide (green channel), DAPI (blue channel). (**H**) The posterior end of a growing worm. (**I**) Old ventral nerve cord in fission zone at the middle stage. (**J**) Fission zone at the late stage. Arrowheds point to the new nerve tracts that are beginning to emerge as a branch of the old ventral nerve cord. The asterisk marks the new developing brain, the red line marks the new developing tail region, and the green line marks the new developing head region within the fission zone. Scale bar in (**A**–**G**), 45 mkm. Scale bar in (**H**–**J**), 50 mkm.

mRNAs of the second *Gsx* homologs, *Nco-Gsx2* and *Plo-Gsx2*, are also found in young segments. Moreover, transcripts are detected in the pygidium. Compared to *Gsx1*, the expression pattern of these genes is more complex (Figure 4A–C and Figure 5A). *Gsx2* is up-regulated in surface cells on lateral sides of young segments and in a larger cellular domain on the ventral side, extending to the epidermal epithelium of young segments and the pygidium, as well as to the terminal part of the ventral nerve cord. In *P. longiseta*, the last domain is less distinct than in *N. communis*. To clarify the possible nature of *Gsx2*-positive cells in *P. longiseta*, we performed immunohistochemical detection of the nervous system using antibodies against FMRF-amide (Figure 5H–J), as well as analysis of the expression of *Plo-pax6* (Figure 5G), a marker of the developing nervous system [37,39]. According to our results, in growing *P. longiseta*, at least some of the *Gsx2*-positive cells are located in the region of the posterior terminal end of the ventral nerve cord and *Plo-pax6* expression. Thus, based on the localization of the *Gsx2* transcripts and expression of the specific neural markers, it can be assumed that *Gsx2* genes are expressed in the integument and nervous system of new segments that arise due to activity in the posterior growth zone.

**Figure 6:**
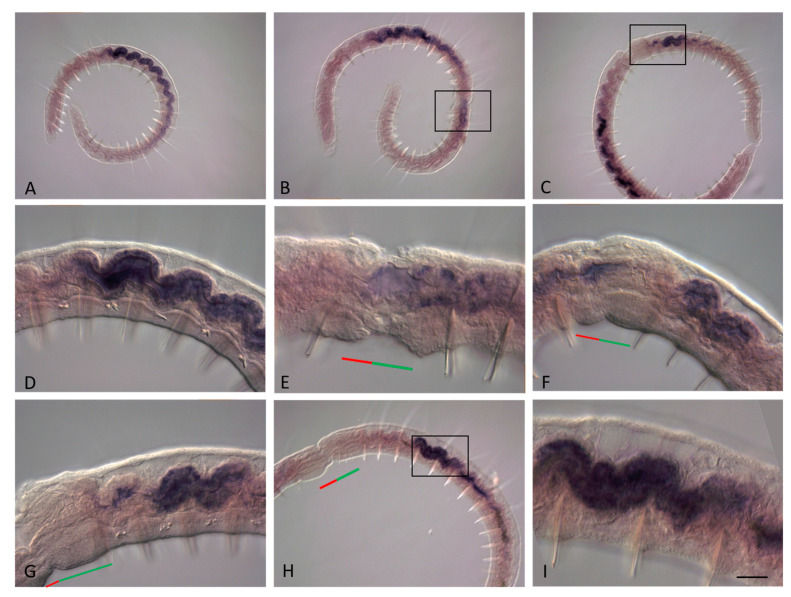
*Nco-Xlox* expression during the growth and asexual reproduction by paratomic fission in *Nais communis*. Except for (**C**), all animals are oriented with the anterior to the left; in (**C**) the anterior is down. Lateral view for all panels. (**A**) *Nco-Xlox* is strongly expressed the middle part of the gut of a growing *N. communis* worm. (**B**) An addition, the domain of expression is visible at the early-middle stage of paratomy. (**C**) Signal in the additional domain of expression becomes stronger at the middle stage of the fission. (**D**) A growing adult worm; enlarged view of the expression pattern in the gut. (**E**) Enlarged view of the boxed region shown in (**B**), deep focal plane. (**F**) Enlarged view of the boxed region shown in (**C**), deep focal plane. (**G**) Another specimen at the middle stage of paratomy. (**H**) Strong expression is observed in the gut cells of the posterior zooid at the late stage of paratomic fission. (**I**) Enlarged view of the boxed region shown in (**H**), deep focal plane. The red line marks the new developing tail region, and the green line marks the new developing head region within the fission zone. Scale bar in (**A**–**C**), 70 mkm; in (**H**), 55 mkm; in (**D**,**G**), 45 mkm; in (**E**,**I**), 35 mkm.

**Figure 7:**
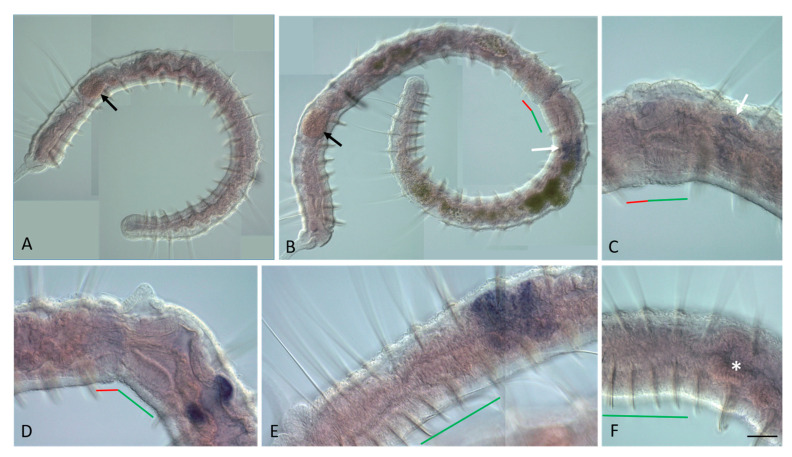
*Plo-Xlox* expression during the growth and asexual reproduction by paratomic fission in *Pristina longiseta*. All animals are oriented with the anterior to the left, lateral view. (**A**) *Plo-Xlox* mRNA is not detected in a growing adult. (**B**) At the late paratomy stage, *Plo-Xlox* (white arrow) is observed in the old gut tissue of the posterior zooid (segments 7,8), which gives rise to the new stomach by tissue remodeling (morphallaxis). (**C**) A very weak expression (arrow) in the gut at the early step of the late fission stage in segment 7. (**D**) At the middle step of the late fission stage, in segment 7, the domain of expression becomes stronger. (**E**) At the end of the late fission stage, expression is observed in segments 7 and 8. (**F**) Expression fades when a stomach (asterisk) becomes morphologically apparent. The red line marks the new developing tail region, the green line marks the new developing head region within the fission zone, and the black arrow marks the stomach. Scale bar, 45 mkm for all panels except (**A**,**B**). Scale bar in (**A**,**B**), 60 mkm.

*Xlox* is expressed in the gut of *N. communis*. The *Nco-Xlox* expression domain marks the midgut (Figure 6A,E) and varies in length from three to six segments in different individuals. Of the analyzed thirty *N. communis* specimens, sixteen had a domain in length of three segments, eight had four segments, three had five segments, and only one had a signal within six segments. A stable anterior and posterior boundary of *Nco-Xlox* expression is also not shown. In different animals, the anterior-most position of expression varies from the ninth to the twelfth segment, being located in the majority in the tenth–eleventh segments. The posterior-most expression is observed at the level of the eleventh–fifteenth segments, but in most cases in the thirteenth or fourteenth segment. At the growth stage of *P. longiseta*, *Plo-Xlox* transcripts were not detected by WMISH (Figure 7A).

**Figure 8:**
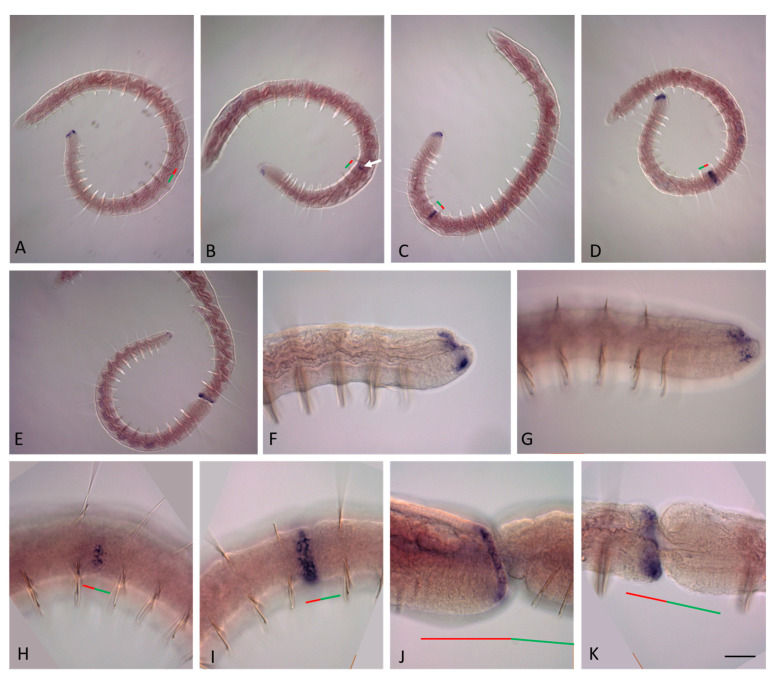
*Nco-Cdx1* expression during the growth and asexual reproduction by paratomic fission in *Nais communis*. Except for (**C**), all animals are oriented with the anterior to the left; in (**C**) the anterior is up. Lateral view for all panels. (**A**) *Nco-Cdx1* is strongly expressed in epidermal cells on the lateral and ventral sides at the posterior end of a growing *N. communis* worm. (**B**) An additional domain of *Nco-Cdx1* expression (arrow) is visible in the fission zone already at the very early stage of paratomy. (**C**,**D**) Expression becomes broader at the middle stage of paratomy. (**E**) At the late stage of fission zone formation, *Nco-Cdx1* mRNA marks the newly formed growth zone and pygidium. (**F**,**G**) Enlarged view of the posterior end of a growing adult, different focal planes. (**H**) Expression of *Nco-Cdx1* at the early stage of paratomy. (**I**) Expression of *Nco-Cdx1* at the middle stage of paratomy. (**J**) Expression of *Nco-Cdx1* in epidermal cells of the new developing tail region of the anterior zooid at the late stage of paratomy. (**K**) Expression of *Nco-Cdx1* in the new developing tail region of the anterior zooid at the late stage of paratomy, deep focal plane. The red line marks the new developing tail region, and the green line marks the new developing head region within the fission zone. Scale bar in (**A**,**B**), 70 mkm; in (**F**–**K**) 45 mkm.

**Figure 9:**
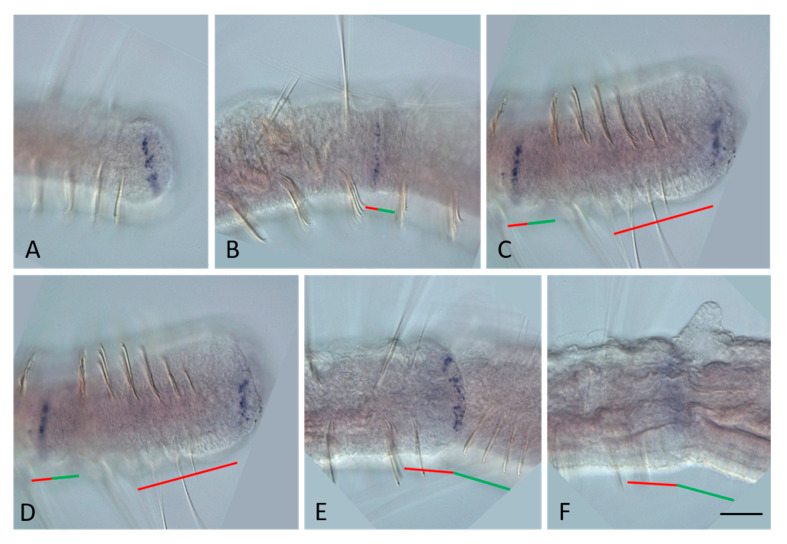
*Plo-Cdx1* expression during the growth and asexual reproduction by paratomic fission in *Pristina longiseta*. All animals are oriented with the anterior to the left, lateral view. (**A**) *Plo-Cdx1* is strongly expressed in epidermal cells on the lateral and ventral sides at the posterior end of a growing *P. longiseta* worm. (**B**) A domain of *Plo-Cdx1* expression is visible in the fission zone at the very early stage of paratomy. (**C**,**D**) Expression becomes stronger during the middle-late stage of paratomy. In the same panels, expression is also observed in an additional fission zone. The posterior zooid was removed during in situ hybridization procedures. (**E**,**F**) At the late stage of fission zone formation, *Plo-Cdx1* mRNA marks the newly formed growth zone and pygidium, different focal planes. The red line marks the new developing tail region, and the green line marks the new developing head region within the fission zone. Scale bar, 45 mkm for all panels.

*Nco-Cdx1* and *Plo-Cdx1* are up-regulated in the epidermal epithelium of the posterior growth zone. The expression domain is ring-shaped (Figure 8A,F,G, and Figure 9A) and, in most specimens, is two to three rows of cells in width.

**Figure 10:**
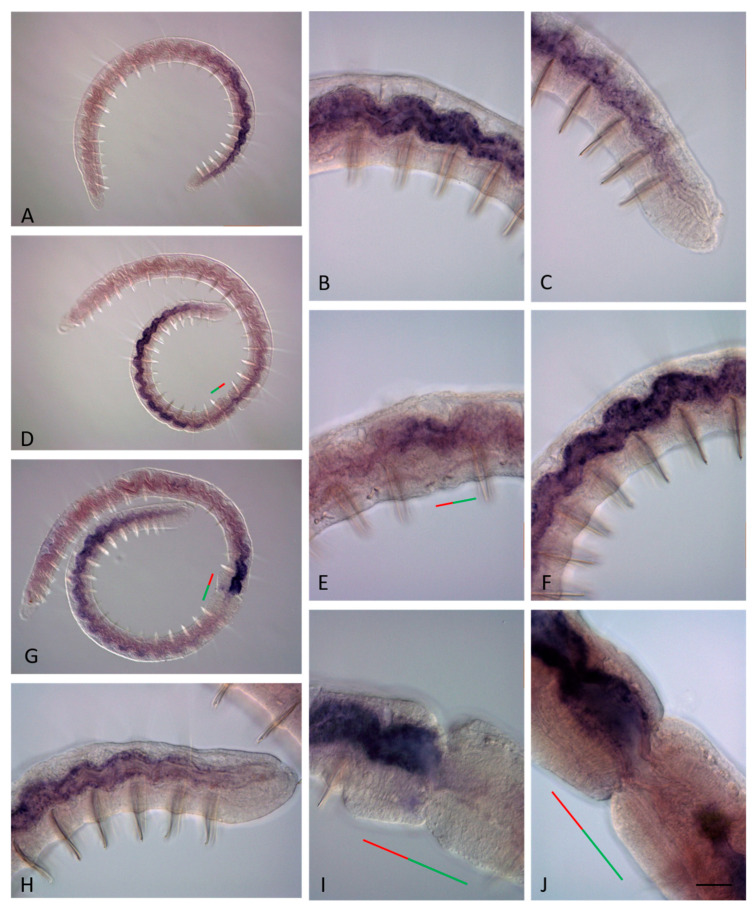
*Nco-Cdx2* expression during the growth and asexual reproduction by paratomic fission in *Nais communis*. All animals are oriented with the anterior to the left, lateral view. (**A**) *Nco-Cdx2* is strongly expressed in the posterior part of the gut of a growing *N. communis* worm. (**B**,**C**) Enlarged view of the anterior and posterior part of *Nco-Cdx2* expression domain, respectively. (**D**) An additional domain of a weak *Nco-Cdx2* expression is visible in the gut within the fission zone at the early stage of paratomy. (**E**) Enlarged view of the early fission zone with a weak expression in the gut. (**F**) Enlarged view of *Nco-Cdx2* expression domain in the posterior zooid at the early fission stage. (**G**) At the middle stage, *Nco-Cdx2* expression becomes stronger in the gut of the new developing tail region. (**H**) Enlarged view of the posterior part of *Nco-Cdx2* expression domain in the gut of the developing posterior zooid at the middle stage. (**I**,**J**) Enlarged view of expression in the gut at the posterior end of the anterior zooid at the middle stage, different specimens. The red line marks the new developing tail region, and the green line marks the new developing head region within the fission zone. Scale bar, 45 mkm for all panels except (**A**,**D**,**G**). Scale bar in (**A**,**D**,**G**), 70 mkm.

**Figure 11:**
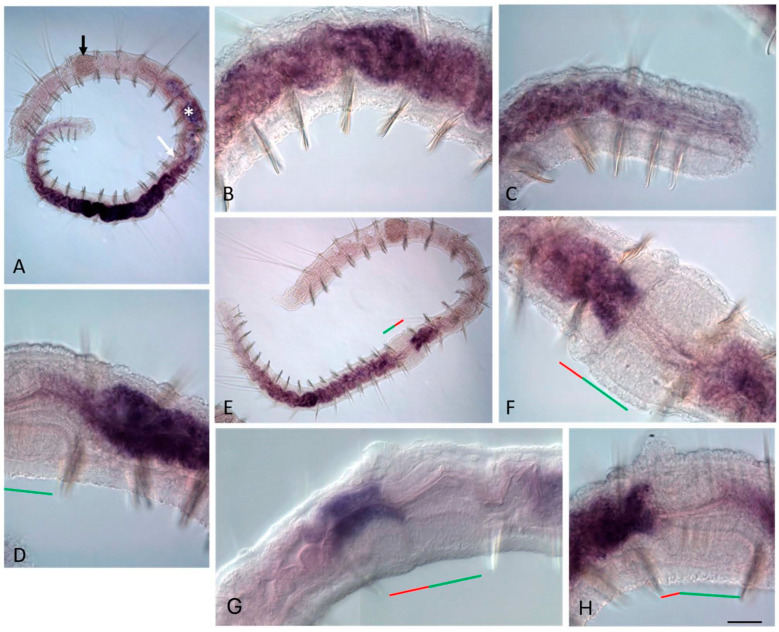
*Plo-Cdx2* expression during the growth and asexual reproduction by paratomic fission in *Pristina longiseta*. All animals are oriented with the anterior to the left, lateral view. In (**F**), a middle stage fission zone is viewed from the ventral view. (**A**) *Plo-Cdx2* is strongly expressed in the posterior part of the gut of a growing *P. longiseta* worm. The *black arrow* marks the stomach, the *white arrow* shows the anterior border of expression, the *asterisk* marks unspecific staining. (**B**,**C**) Enlarged view of the anterior and posterior part of the *Plo-Cdx2* expression domain, respectively. (**D**) Anterior part of the developing posterior zooid at the middle stage, lateral view. (**E**) An addition domain of a *Plo-Cdx2* expression is visible in the gut anterior and in the fission zone at the middle stage of paratomy. (**F**) Enlarged view of *Nco-Cdx2* expression domain in the fission zone of the animal shown in (**E**). (**G**,**H**) Enlarged view of expression in the gut at the posterior end of the anterior zooid and the anterior part of the posterior zooid at the late stage, different specimens. The *red line* marks the new developing tail region, and the *green line* marks the new developing head region within the fission zone. Scale bar, 45 mkm for all panels except (**A**,**E**). Scale bar in (**A**,**E**), 70 mkm.

*Nco-Cdx2* and *Plo-Cdx2* genes are expressed in posterior part of the gut (Figure 10A–C and Figure 11A–C), and their signal has a distinct anterior–posterior gradient. In *N. communis*, the highest level of transcripts forms on the fifth or sixth segment, counting from the tail end. As in the case of *Nco-Xlox*, the boundaries and length of the *Nco-Cdx2* expression domain vary, and even more strongly. Of the forty analyzed specimens, most of all were animals with an expression domain of seven segments in length. On the other hand, this group of objects made up only a quarter of the sample, and the nearest values of the domain length (from six to ten segments) were also quite common. The minimum and maximum values amounted to three and thirteen segments, respectively, and are represented by single specimens. As mentioned above, the signal decreases posteriorly, which makes it impossible to determine the district posterior boundary of expression, but according to the visible signal, it varies from the pygidium to the fourth segment ahead. In animals of different lengths, the anterior boundary of *Nco-Cdx2* expression is at the level of the fourteenth to twenty-seventh segment from the anterior end. Variability of *Cdx2* expression boundaries along the anteroposterior axis was also noted for *P. longiseta*.

### 3.3. ParaHox Gene Expression in the Asexually Reproducing Animals, N. communis and P. longiseta

At the beginning of the fission zone development, mRNA of *Plo-Gsx1* and *Nco-Gsx1* persists at low levels in single surface cells on the ventral side of young terminal segments in both *P. longiseta* and *N. communis* (Figure 2B). Later, the expression becomes even weaker or disappears completely for a while. No signal was detected within the paratomy zone at the early and middle stages (Figure 2E and Figure 3B). At the late stage, a weak signal appears de novo in a few ventral cells of the somatogenic part of the fission zone, i.e., the future posterior end of the anterior zooid (Figure 2E,F and Figure 3C). Thus, expression in the anterior zooid occurs in the same area, and probably in the same cells, as in growing adults.

Although the *Gsx2* mRNA is not detected at the very early steps, when the fission zone cannot be recognized at the morphological level, in contrast to *Gsx1*, it is expressed in a new domain already at the early stage of asexual reproduction (Figure 4D–I and Figure 5B–F). In *N. communis*, *Nco-Gsx2* transcripts are observed in several surface cells on the ventral side of the somatogenic part of the fission zone. Later, expression increases, and three definitive expression domains appear: two bilateral and a larger ventral one; the same as those described at the tail end of growing worms (Figure 4F–I). Expression on the ventral side of the pygidium and young segments at the posterior end of the posterior zooid persists throughout the fission process (Figure 4J). Compared to *Nais*, in *Pristina*, *Plo-Gsx2* expression, although at a low level, is observed not only on the ventral but also on the lateral sides of the early paratomy zone (Figure 5B). Later, expression becomes stronger and more characteristic of the posterior end of the growing animals (Figure 5C–F). Moreover, *Plo-Gsx2* shows a similar expression pattern within the additional fission zone (Figure 5E,F), if it develops.

During asexual reproduction, the patterns of *Xlox* gene activity are distinctly different in the studied species (Figure 6B–I and Figure 7B–F). In *N. communis*, an additional expression domain of the *Nco-Xlox* gene appears de novo in the gut of the posterior zooid at the early-middle fission stage (Figure 6B,E). Transcripts are found first in the gut of the segment within which the paratomy zone is formed, as well as in the gut of the adjacent segment. Later, during the development of the fission zone, this two-segment expression domain becomes gradually shifted posteriorly, eventually occupying a position similar to that in growing animals. Expression disappears first in the fission zone, and then in the segments adjacent behind. Thus, the anterior boundary of the *Nco-Xlox* additional expression domain is shifted posteriorly, and in later stages, the transcripts are detected in the ninth to tenth segments of the posterior zooid (taking into account four developing head segments) (Figure 6F–H). In the posterior segments of the anterior zooid, *Nco-Xlox* mRNA is not detected at the late stage of paratomy.

In *P. longiseta* worms, as described above, *Xlox* is not expressed at the growth stage. The first signs of gene activity are observed at the beginning of the late stage of asexual reproduction. In this stage, *Plo-Xlox* mRNA appears de novo in the intestinal tissues of the most anterior old segment of the posterior zooid, adjacent to the paratomy zone (in the future, segment 7, taking into account the developing six head segments in the cephalogenic part of fission zone) (Figure 7C). The level of expression is very low. Then the signal becomes very strong and the expression domain extended posteriorly to the gut of the next segment (Figure 7D,E). After the ending of the fission process, this part of the intestine will form a new stomach of the posterior zooid by tissue remodeling (morphallaxis). *Plo-Xlox* expression disappears when the stomach begins to be morphologically recognizable (Figure 7F).

During paratomy, both *Nco-Cdx1* and *Plo-Cdx1* are expressed before the other ParaHox genes (Figure 8B–K and Figure 9B–F). *Cdx1* transcripts were detected by in situ hybridization at the earliest stages of paratomy. Expression begins in the segment in which the fission zone will be formed, even before the morphological signs of the fission zone become apparent. The signal is not observed exactly in the middle of the segment, but is shifted forward, which makes it possible to determine the border between the somatogenic and cephalogenic parts of the developing paratomy zone. At the early fission stage, the signal increases in the cells that form a band around most of the circumference of the fission zone, marking the posterior border of the anterior zooid (Figure 8B,H–K, and Figure 9B). Domain of expression extends over two rows of cells in width. At the middle and late fission stages, the gene continues to be expressed in the posterior terminal part of the anterior zooid (Figure 8H–K and Figure 9C–F). *Cdx1* persists in expression in the posterior growth zone of the posterior zooid throughout the entire period of growth and asexual reproduction in both *N. communis* and *P. longiseta*.

In both species, at the early-middle paratomy stage, *Cdx2* transcripts are found as an additional expression domain in the gut in the segment where the fission zone is formed (Figure 10D–J and Figure 11D–H). At an early stage, *Nco-Cdx2* and *Plo-Cdx2* mRNA occur in the intestinal tissue within the entire fission zone in *N. communis* and *P. longiseta*, respectively. Later, it is limited to the gut tissue located in the somatogenic part of the fission zone. At later stages, expression increases and is observed in the gut of the somatogenic part and the segment adjacent to the fission zone. The initial signal in the posterior gut of the posterior zooid is observed at the early stages, but decreases at the middle stage and later disappears, persisting only in the gut of the trunk segments.

## 4. Discussion

### 4.1. ParaHox Genes in Naidids N. communis and P. longiseta

The identification of five ParaHox genes in both studied species, *N. communis* and *P. longiseta*, provides further evidence for the expansion of homeobox-containing genes, particularly the ANTP class genes, in the oligochaete lineage. For example, in the genome of another oligochaete, *Eisenia foetida*, in addition to numerous Hox gene homologs (28 in total), three *Gsx* paralogs, two *Xlox (Pdx)* paralogs, and four *Cdx* paralogs were found [52]. Multiple gene duplications of the ANTP class were also discovered in the genome of the leech *H. robusta*. Specifically, twenty-eight *Cdx* homologs were described [52,53]. This finding is intriguing because leeches and oligochaetes are considered a monophyletic group (Clitellata). In the genome of the polychaete *C. teleta*, which belongs to the Sedentaria group, which includes clitellate annelids, the presence of two *Xlox (Pdx*) paralogs (although only one was studied in terms of cloning and expression analysis by [16]) is described, while *Gsx* and *Cdx* are represented by single genes [52]. Thus, there is a tendency for ParaHox gene duplications in the annelid lineage, especially for *Gsx* and *Cdx*, whose paralogs, as shown in this study, have distinct expression domains and may perform different functions. As it is known, ParaHox genes are organized into complete clusters in the genomes of many animal species [1,3,12,53,54,55]. It is difficult to determine the origin of the five studied homologs since the genomes of our species have not been sequenced, and it is unknown whether an intact ParaHox cluster exists or whether it has disintegrated. Two scenarios can be suggested for the emergence of this particular set of genes. It is possible that a duplication of the entire three-gene cluster occurred, followed by the loss of one of the *Xlox* orthologs. Alternatively, the five genes may have arisen through two independent duplications of *Gsx* and *Cdx*. Both scenarios require an equal number of evolutionary events, so neither can be excluded based on the principle of parsimony.

### 4.2. Expression of ParaHox Genes in Growing adult N. communis and P. longiseta

**Figure 12:**
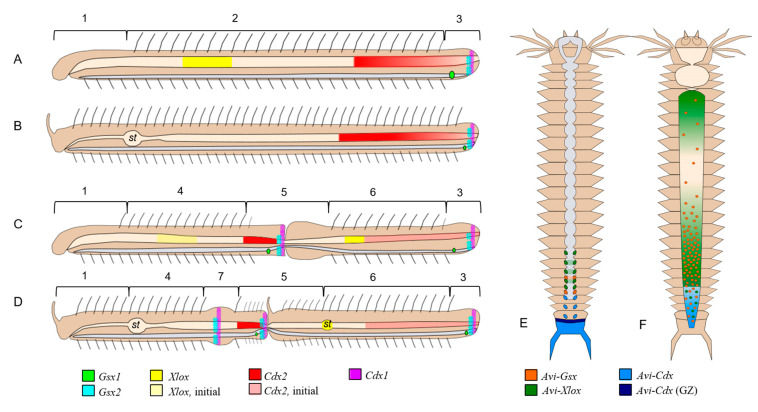
ParaHox gene expression during postembryonic development in annelids. (**A**–**D**) A scheme of ParaHox gene expression during the growth (**A**,**B**) and asexual reproduction by paratomic fission (**C**,**D**) in *Nais communis* (**A**,**C**) and *Pristina longiseta* (**B**,**D**). Animals are oriented with the anterior to the left. 1—head segments, 2—trunk segments, 3—young segments, posterior growth zone and pygidium, 4—trunk segments of the anterior zooid, 5—fission zone, 6—trunk segments of the posterior zooid, 7—additional fission zone, and *st*—stomach. (**E**,**F**) A scheme of ParaHox gene expression in growing juvenile *A. virens*, based on data from [15]. The anterior is up. (**E**) Expression in the pygidium, posterior growth zone, and ventral nerve cord. (**F**) Expression in the digestive system. GZ—posterior growth zone. The nervous system is shown in grey in all schemes.

In this study, we found that ParaHox genes are expressed in the gut (*Nco-Xlox*, *Nco-Cdx2*, and *Plo-Cdx2*) as well as in the tissues in young segments and pygidium (*Gsx1*, *Gsx2*, and *Cdx1* in both species) of the growing adult worms (Figure 12A–D). In contrast, in *P. longiseta*, *Plo-Xlox* is not detected at the growth stage by WMISH.

Interestingly, in both species, duplicated *Gsx* homologs show unexpected expression at the posterior end of the animals (Figure 2, Figure 3, Figure 4, Figure 5 and Figure 12). According to knowledge on protostomes, the homologs of this gene should be active in the anterior parts of the body, in the gut or nervous system. Undoubtedly, we should keep in mind that the postembryonic expression pattern may differ from the embryonic one. In *A. virens* (formerly *Nereis virens*) juveniles, *Gsx* expression was shown in the midgut [15] (Figure 12F). In oligochaetes studied in this work, both paralogs, *Gsx1* and *Gsx2*, are up-regulated in the surface cells of young (posterior) segments (Figure 2, Figure 3, Figure 4 and Figure 5), which do not yet have formed chaetae, and the *Gsx2* expression domain is also observed in the pygidium. Nevertheless, there is a similarity between *Gsx* expression in juvenile *A. virens* and species investigated in this study. In *A. virens*, all three *ParaHox* genes were shown to be involved in the patterning of neuromeres formed due to activity of the growth zone [15] (Figure 12E). In *N. communis* and *P. longiseta*, at least one of the *Gsx* homologs, *Gsx2*, is expressed in the terminal part of the ventral nerve cord and may be active in the nervous system of newly formed segments. *Nco-Gsx1* and *Plo-Gsx1* have a weaker expression in single superficial cells on the ventral side of young segments closer to the border of the last segment bearing setae. In naidids, the exact nature of the *Gsx1*-positive cells is unknown, but their belonging to the nervous system is not excluded. In any case, the results of our study suggest the functional diversification of *Gsx* duplicates in both *N. communis* and *P. longiseta*. These genes may play an important role in patterning and specification of the nervous system, epidermal cells of the young trunk (i.e., postlarval [56]) segments, and pygidium (Figure 12A–D).

The results of our study support the ancestral role of *Gsx* in the patterning of the nervous system in both clitellate and non-clitellate annelids. In the laboratory culture, both species studied in this work reproduce only asexually. Other species that reproduce sexually should be investigated to find if *Gsx* is involved in the specification of the anterior anlagen, including the gut, at the embryonic stages in oligochaetes.

Expression of *Xlox* gene differs very dramatically in adult growing naidids. While *Plo-Xlox* is down-regulated in *P. longiseta*, *Nco-Xlox* shows a more predictable expression pattern in the midgut with a domain of three to four segments in length (Figure 6, Figure 7 and Figure 12A–D). For juvenile *A. virens*, the midgut expression of *Xlox* is described as three domains of different signal intensity: an anterior–posterior gradient starting at the anterior border of the midgut, a double gradient in the posterior part of the midgut, and individual cells in the hindgut [15]. In *N. communis*, the *Nco-Xlox* domain is significantly shorter in length but shows variable positions within the midgut (Figure 6). Moreover, *Nco-Xlox* expression was not detected at all in the nervous system of the worm, in contrast to *A. virens* (Figure 12E,F).

Like *Gsx*, *Cdx* duplicates demonstrate expression in the posterior part of the body but with different patterns for *Cdx1* and *Cdx2* (Figure 8, Figure 9, Figure 10, Figure 11 and Figure 12). In both naidid species, *Cdx2* transcripts are detected in the posterior part of the gut, while *Cdx1* is found in the epidermal epithelium of the posterior end of the body in the form of a ring of one–two cell rows in width. Localization of the *Cdx1*-positive cells correspond to the ectodermal part of the growth zone [15,34,35,43,51,57,58]. The ortholog of this gene in *P. dumerilii* has a similar expression pattern in juvenile worms during posterior regeneration [26,59]. In addition to *Cdx*, expression of other genes encoding transcription factors, such as *Hox3* and *Eve* [26,60,61,62], was shown in the growth zone, and these genes are of future interest for studying the patterning and functioning of the growth zone in annelids.

Expression pattern of the second paralogs of *Cdx*, *Nco-Cdx 2* and *Plo-Cdx 2* looks like an anterior–posterior gradient within the hindgut (Figure 10, Figure 11 and Figure 12). Although in *P. dumerilii*, *Cdx* does not display a similar expression domain [26], in another nereidid, *A.virens*, *Cdx* is expressed both in the growth zone and in the hindgut [15] (Figure 12E,F), combining the spatial domains identified for *Cdx1* and *Cdx2* in naidid oligochaetes (Figure 12A–D).

In *C. teleta* juveniles, *Cdx* is also expressed in the hindgut; however, it shows specific expression in the pharynx and ventral nerve cord. Interestingly, expression is not detected in the growth zone in *C. teleta* [16]. Based on the greater phylogenetic similarity between the capitellid and naidids belonging to Sedentaria [63], it may seem that conclusions should be drawn primarily from comparison with *C. teleta*, and not with nereidids, but the unusual pattern of *Cdx* expression in *C. teleta* gives every reason to believe that this gene in capitellid has undergone evolutionary changes, possibly in its regulation, and therefore its expression pattern has strongly diverged from the ancestral one [16].

Considering all of the above, it can be assumed that *Cdx* in the postlarval development of annelids is characterized primarily by expression in the hindgut and growth zone. A similar double pattern was preserved in *A. virens*, but specific domains in the growth zone and hindgut were lost in *C. teleta* and *P. dumerilii*, respectively. In *N. communis* and *P. longiseta*, these functions were taken over by duplicated homologs (Figure 12). It should also be noted that none of the *N. communis* and *P. longiseta Cdx* homologs are expressed in the nervous system of worms, in contrast to the *A. virens* ortholog [15].

### 4.3. Expression of ParaHox Genes in Asexually Reproducing N. communis and P. longiseta

The expression of each gene analyzed in this study changes noticeably during paratomic fission in *N. communis* and *P. longiseta* (summarized in Figure 12A–D). In all cases, these changes resulted in the appearance of additional expression domains in the developing fission zone and zooids and/or in adjustment of the initial mRNA signal along the anterior–posterior axis. Thus, the genes expressed in young segments and pygidium (*Gsx1*, *Gsx* 2, and *Cdx1*) become transcribed in a similar region of the somatogenic part of the fission zone. *Nco-Xlox* mRNA initially presents in the midgut and appears during paratomy in the gut in the posterior zooid, first in the region adjacent to the fission, and later it occurs in the definitive region at a distance of eight segments from the anterior end of the new individual. *Plo-Xlox* expresses de novo in the gut tissue in the old segments, where a new stomach of the posterior zooid starts to be formed by tissue remodeling. *Cdx2* transcripts appear in the hindgut of the anterior zooid in both species.

Expression of “intestinal” genes shows the features of molecular morphallaxis [39,40]. The *Nco-Xlox* domain, which becomes apparent in the gut of the paratomy zone segment, gradually shifts posteriorly to the future midgut region of the posterior zooid (Figure 12C). The initial domain gradually disappears in the most posterior part of the anterior zooid. In contrast to *N. communis*, *P. longiseta* has a stomach that develops in the posterior zooid by gut remodeling. Interestingly, *Plo-Xlox* expression occurs and then disappears before the morphologically visible stomach formation. Initial expression domain of *Cdx2* orthologs in the hindgut is also changed during fission and then re-established along the anterior–posterior axis in the daughter zooids. During asexual reproduction by architomy (parent organism physically separates into fragments before the new head and the new tail develop) in oligochaete *Enchytraeus japonensis,* similar expression rearrangements are also known for *Tuba*, *mino*, and *horu* genes [64].

*Gsx1*, *Gsx2*, and *Cdx1* orthologs also demonstrate dynamic expression during paratomy. *Cdx1* genes are the earliest activated ParaHox genes during asexual reproduction in both *N. communis* and *P. longiseta*. These genes are expressed at the established posterior end in the anterior zooid before any morphological signs of the fission zone formation. As discussed above, *Cdx1* may be involved in the growth zone formation and development of new segments in growing individuals. Accordingly, during paratomy, it probably performs a similar function and determines the early specification of a new growth zone in the anterior zooid. Considering the early *Cdx1* expression, it could be assumed that this gene is critical for the process of specification and differentiation of the fission zone. On the other hand, other genes are also known to be activated at such an early stage of paratomy; for example, *Otx* and *Six3* in *P. longiseta* [35]. All these genes can be involved in specifying the anterior boundary of the prospective somatogenic part of the fission zone [35,41]. At the early stages, they, like *Nco-Cdx1* and *Plo-Cdx1*, are expressed in the surface epithelium, but later the *Plo-Otx*-positive cells are found in the deep blastemal cell mass.

In *N. communis* and *P. longiseta*, *Gsx* homologs are up-regulated in the posterior part of both the growing worms and the presumptive posterior end of the zooids. The absence of *Gsx* expression during mouth formation in this case can be explained by a difference in the genetic program controlling this morphogenesis during asexual reproduction and embryogenesis. It is even more obvious if we take into account the fact that, during paratomy, it is not the de novo formation of the mouth, but rather the morphallactic reorganization of the intestine in the cephalogenic part of the fission zone [34,35,37,38,40].

How ParaHox genes are regulated in annelids remains an open question. One of the candidates for upstream regulators of ParaHox genes is Wnt signaling. Participation of Wnt in the formation of the gut was shown at the larval stages in *P. dumerilii* [65]. In addition, there are works that demonstrate the regulation of not only *Cdx*, but also other ParaHox genes by Wnt signaling [66,67]. Retinoic acid and members of the Hedgehog signaling pathway may be other candidates for upstream regulators of ParaHox genes [68,69]. A direct relationship between Hedgehog signaling and ParaHox genes has not been described, but its activity is known in the developing gut in *H. robusta* and *C. teleta* [70,71].

### 4.4. Signs of Colinearity in the Expression of ParaHox Genes in Growing and Asexually Reproducing N. communis and P. longiseta

The question about the colinearity of ParaHox and Hox gene expression is one of the most important [2,3,4]. In our case, for both *N. communis* and *P. longiseta*, genomes are currently not sequenced, and there is nothing known about the genomic organization of ParaHox genes in these species.

In growing *N. communis* worms, signs of spatial colinearity are observed for the expression of *Nco-Xlox* and *Nco-Cdx2* in the gut. The first is expressed in the midgut, and the second in the posterior part of the gut (Figure 12). During paratomic fission, these genes also show spatially colinear expression in the daughter zooids. The specificity in this case is that the new *Xlox* expression domain will be located ahead of the old intact *Cdx2* domain, which weakens and gradually disappears. Similarly, a new *Nco-Cdx2* expression domain in the anterior zooid will be located behind the intact *Xlox* expression domain, which also weakens and disappears. In *P. longiseta*, *Plo-Xlox* and *Plo-Cdx2* are expressed with spatial colinearity only in the posterior zooid. Other Parahox are up-regulated in the nervous system and integuments of the posterior part of the body, in young segments and pygidium. *Cdx1* demonstrates spatial colinearity of its expression; however, *Gsx* paralogs may lack this feature since their expression is expected to be more anteriorly located. Without genomic data, it is impossible to make a correct conclusion about the possible spatial colinearity of *Gsx* paralog expression.

The issue of temporal colinearity is more complex. During paratomy, expression of *Nco-Cdx1* is initiated before others. Apparently, *Nco-Gsx2* starts next in the area of the somatogenic part of the fission zone. After that, the “intestinal” genes *Xlox* and *Cdx2* are activated, and at the last stage *Gsx1* is activated. Thus, during asexual reproduction, ParaHox genes show expression with signs of spatial colinearity in the gut, but temporal colinearity is broken.

## Data Availability

mRNA sequences of *Nco-Gsx1*, *Nco-Gsx2*, *Nco-Xlox*, *Nco-Cdx1*, *Nco-Cdx2*, *Plo-Gsx1*, *Plo-Gsx2*, *Plo-Xlox*, *Plo-Cdx1*, *Plo-Cdx2* and a 5′ partial cds of *Plo-Cdx2* are deposited in GenBank with the accession numbers OR050790–OR050799 and JQ685130, respectively. The GenBank accession number of *Plo-pax6* mRNA sequence is OR258068.

## Supplementary Materials

The following supporting information can be downloaded at: https://www.mdpi.com/article/10.3390/genes14071501/s1, Table S1: Primer sequences used to clone fragments of the *Plo-* and *Nco-ParaHox* genes presented in the paper; Table S2: GenBank accession numbers for sequences used for Gsx, Xlox, and Cdx amino acid alignments; and Figure S1: CLUSTAL W 2.0 multiple alignment of ParaHox sequences used for phylogenetic analysis (Figure 1).
