# Supplementary material for: Spatial Colinear but Broken Temporal Expression of Duplicated ParaHox Genes in Asexually Reproducing Annelids, Nais communis and Pristina longiseta
Source: Genes (Basel). 2023 Jul 22;14(7):1501. doi: 10.3390/genes14071501 (PMC10379933; doi:10.3390/genes14071501)
Supplement: Supplementary file 1 [file genes-14-01501-s001.zip › genes-2459219-supplementary.pdf]

Supplementary Materials

Table S1. Primer sequences used to clone fragments of the *Plo*-and *Nco-Parahox* genes presented in the paper

***Plo-Gsx*, *Plo-Xlox*, and *Plo-Cdx* short fragments**		
forward		5'-GCBCARYTNGTHGARYTVGARAARG-3'
forward		5'-GGNAARACNMGNACNAARGAYAARTAYMG-3'
forward		5'-CARYTKYTDGARYTHGARMGRG-3'
reverse		5'-CKNCKRTTYTGRAACCA-3'
***Plo-Gsx*, *Plo-Xlox*, and *Plo-Cdx* RACE fragments**		
Plo-Gsx 3'RACE	forward	5'-GTCACTTTCGGACGAACGAGTACC-3'
	forward nested	5'-GGTCGAACGCCGCGCATCGAAATG-3'
Plo-Gsx 5'RACE	reverse	5'-GATTTGCTGTTCAGACAGGTTTAAAGCG-3'
	reverse nested	5'-CCATTTCGATGCGCGGCGTTCGAC-3'
Plo-Gsx 3'RACE	forward	5'-GCTAGAGCTTGAGCGTCAGTTCAAC-3'
	forward nested	5'-CCAACATGTACCTCTCGAGATTGC-3'
Plo-Gsx 5'RACE	reverse	5'-GATCTGTTTTTCGGAAAGCTGGAGGCAG-3'
	reverse nested	5'-CTGCAATCTCGATCCGGCGCAATCTC-3'
Plo-Xlox 3'RACE	forward	5'-GCTACTTGAGCTCGAGAAAGAATTTCAC-3'
	forward nested	5'-CTACGACAAATATATATCCAGAGCTCGAC-3'
Plo-Xlox 5'RACE	reverse	5'-GTGACGCTCTGTCAGTCGAAGAACTTTG-3'
	reverse nested	5'-GGCCATTTCCAGACGTCGAGCTC-3'
Plo-Cdx 3'RACE	forward	5'-CGCAGTTAGTCGAGTTCGAAAAGGAA-3'
	forward nested	5'-CAATTCAACGTAAGGCTGAGCTGGCTGG-3'
Plo-Cdx 5'RACE	reverse	5'-CGGCTGTTTGGAACCAGATCTTAACTTG-3'
	reverse nested	5'-CTGTCTGAGAGTCCGATAGTGCCAGCC-3'
Plo-Cdx 3'RACE	forward	5'-CCAGCTCGTCGAGTTGGAGAAGG-3'
	forward nested	5'-CCAGTACATCACAATACAACGCAAAGC-3'
Plo-Cdx 3'RACE	reverse	5'-GGTTTTGGAACCAGATCTTGACCTGACGG-3'
	reverse nested	5'-GGTCTGATAGACCAATATGTCCAGCAAG-3'
***Plo-Gsx*, *Plo-Xlox*, and *Plo-Cdx* fragments used as templates for RNA probes ***		
Plo-Gsx1	forward	5'-GAAGATGCCCGACATTCGTTCTGTTG-3'
	reverse	5'-GTCCCGTGTATAAGTTGCGATAATGC-3'
Plo-Gsx2	forward	5'-GAATTATCCAGCCGTCGACTGCCATC-3'
	reverse	5'-CAGTCCACGATGTTCTTGCCGACAAC-3'
Plo-Xlox	forward	5'-CCGTCACTCAGAGGATTGATGGAAAAC-3'
	reverse	5'-GACGTTGTGAAGTGGGCGGATGTG-3'
Plo-Cdx1	forward	5'-GCAATCGTTCGTTACCTGCCGCTG-3'
	reverse	5'-CACTCTATCGTCAGGCAATGTAACC-3'

*Previously cloned 5'partial CDS fragment of *Plo- Cdx2* (1507 bp, GenBank JQ685130) was used as a template for *Plo-Cdx2* RNA probe.

***Nco-Gsx*, *Nco-Xlox*, and *Nco-Cdx* fragments used as templates for RNA probes**		
Nco-Gsx1	forward	5'-CAGTCATTCCTCGTCGATTCACTCC-3'
	reverse	5'-GGTGGTTGTTGCTGTAGCTGATGTC-3'
Nco-Gsx2	forward	5'-CTGCCCTGTCGTCTCTGGTAGCAC-3'
	reverse	5'-GTAGTGTGGTGGACGAATCAGTTGC-3'
Nco-Xlox	forward	5'-GACTGGTTACAGCAGCAGGGACG-3'
	reverse	5'-CAGCAACTCCATCAATCTCCGAC-3'
Nco-Cdx1	forward	5'-GGTCACGGGTCAACTTATAGTCATC-3'

	reverse	5'-GAACCAAATCTTGACTTGACGCTCG-3'
Nco-Cdx2	forward	5'-GGTACACCATATTGACGATAGCATCAG-3'
	reverse	5'-CCGTTCCGCTTGATGATCCACTAC-3'

Table S2. GenBank accession numbers for sequences used for Gsx, Xlox, and Cdx amino acid alignments

Phylum	Gene name in tree	Species	GenBank #
Annelida	Cte_Antp	(*outgroup*) *Capitella teleta,* Antp Hox	EU196547
	Nco-Gsx1	*Nais communis*	OR050790
	Nco-Gsx2	*Nais communis*	OR050791
	Plo-Gsx1	*Pristina longiseta*	OR050795
	Plo-Gsx2	*Pristina longiseta*	OR050796
	Avi_Gsx	*Alitta virens*	ABB59695.1
	Cte_Gsx	*Capitella teleta*	AAZ23124.1
	Pdu_Gsx	*Platynereis dumerilii*	ACH87538.1
	Nco-Xlox	*Nais communis*	OR050792
	Plo-Xlox	*Pristina longiseta*	OR050797
	Cte_Xlox	*Capitella teleta*	AAZ95509.1
	Hro_Xlox	*Helodbella robusta*	XP_009020396.1
	Pdu_Xlox	*Platynereis dumerilii*	ACH87541.1
	Nco-Cdx1	*Nais communis*	OR050793
	Nco-Cdx2	*Nais communis*	OR050794
	Plo-Cdx1	*Pristina longiseta*	OR050798
	Plo-Cdx2	*Pristina longiseta*	OR050799
	Avi_Cdx	*Alitta virens*	AAN11403.2
	Cte_Cdx	*Capitella teleta*	AAZ95508.1
	Pdu_Cdx	*Platynereis dumerilii*	ACH87546.1
	Pex_Gsx	*Perionyx excavatus*	AAX09760.1
Mollusca	Aca_Gsx	*Aplysia californica*	XP_005093203.2
	Aen_Gsx	*Antalis entalis*	ALM30865.1
	Sof_Gsx	*Sepia officinalis*	ALC76021.1
	Ema_Xlox	*Elysia marginata*	GFR99919.1
	Pma_Xlox	*Pecten maximus*	XP_033742737.1
	Sof_Xlox	*Sepia officinalis*	AHY00652.1
	Pvu_Cdx	*Patella vulgata*	CAD57266.1
Insecta	Tca_Gsx	*Tribolium castaneum*	NP_001034494.1
	Tca_Cdx	*Tribolium castaneum*	XP_008191732.1
Echinodermata	Spu_Gsx	*Strongylocentrotus purpuratus*	XP_784486.3
	Spu_Xlox	*Strongylocentrotus purpuratus*	XP_003728715.2
	Spu_Cdx	*Strongylocentrotus purpuratus*	XP_789158.3
Hemichordata	Sko_Gsx	*Saccoglossus kowalevskii*	XP_002732050.2
	Sko_Xlox	*Saccoglossus kowalevskii*	XM_002741106.1
	Sko_Cdx	*Saccoglossus kowalevskii*	NP_001158415.1
Chordata	Mmu_Gsx1	*Mus musculus*	NP_032204.1
	Xla_Gsx1	*Xenopus laevis*	XP_018103198.1
	Mmu_Gsx2	*Mus musculus*	NP_573555.1
	Xla_Gsx2	*Xenopus laevis*	XP_018098177.1
	Bfl_Xlox	*Branchiostoma floridae*	XP_035682017.1
	Mmu_Xlox	*Mus musculus*	NP_032840.1
	Xla_Xlox	*Xenopus laevis*	XP_018103197.1
	Bfl_Cdx	*Branchiostoma floridae*	XP_035680992.1

	Mmu_Cdx1	*Mus musculus*	AAA37412.1
	Mmu_Cdx2	*Mus musculus*	NP_031699.2
	Mmu_Cdx4	*Mus musculus*	NP_031700.1
	Xla_Cdx1	*Xenopus laevis*	XP_018105758.1
	Xla_Cdx2	*Xenopus laevis*	AAI46638.1
	Xla_Cdx4	*Xenopus laevis*	AAH55999.2
Cnidaria	Nvi_Gsx	*Nematostella vectensis*	AAD39349.1
	Nvi_ParaHox	*Nematostella vectensis*	ACT36590.1

Figure S1. CLUSTAL W 2.0 multiple alignment of ParaHox sequences used for phylogenetic analysis (Figure 1)

```
Cte_Antp        QFGPERKRGRQTYTRYQTLELEKEFHFNRYLTRRRRIEIAHALCLTERQIKIWFQNRRMKWKKEX
Nvi_ParaHox     SSAQVRSRARTAYTASQQLELEKEFLYSRYITRTRRKELANTLDLSEKHIKIWFQNRRMKKKKTD
Cte_Gsx         ESSDAVKRMRTAFSSTQLLELEREFASNMYLSRLRRIEIATYLSLSEKQVKIWFQNRRVKFKKEX
Pdu_Gsx         MEDANGKRIRTAFTSTQLLELEREFSSNMYLSRLRRIEIATYLNLSEKQVKIWFQNRRVKYKKEG
Avi_Gsx         IEDANGKRIRTAFTSTQLLELEREFSSNMYLSRLRRIEIATYLNLSEKQVKIWFQNRRVKYKKEG
Sko_Gsx         NLXTSCKRIRTAFTSTQLLELEREFASNMYLSRLRRIEIATYLNLSEKQVKIWFQNRRVKHKKEG
Spu_Gsx         QIRESSKRIRTAFTSTQLLELEREFAANMYLSRLRRIEIATYLNLSEKQVKIWFQNRRVKYKKEG
Nvi_Gsx         XXXSRSKRIRTAYTSMQLLELEKEFSQNRYLSRLRRIQIAALLDLSEKQVKIWFQNRRVKWKKDK
Tca_Gsx         XXXASSKRIXTAFTSTQLLELEREFASNMYLSRLRRIEIATCLRLSEKQVKIWFQNRRVKYKKED
Aca_Gsx         DLXPSSKRMRTAFTSTQLLELERAFGTNMYLSRLRRIEIATCLNLSEKQVKIWFQNRRVKHKKEG
Xla_Gsx1        QLXPSSKRMRTAFTSTQLLELEREFASNMYLSRLRRIEIATYLNLSEKQVKIWFQNRRVKHKKEG
Aen_Gsx         DLXISSKRIRTAFTSTQLLDLEREFNSNMYLSRLRRIEIATYLNLSEKQVKIWFQNRRVKYKKEE
Sof_Gsx         XXXQSSKRIRTAFTSTQLLELEREFASNMYLSRLRRTEIATYLNLSEKQVKIWFQNRRVKYKKEG
Mmu_Gsx1        QLXPSSKRMRTAFTSTQLLELEREFASNMYLSRLRRIEIATYLNLSEKQVKIWFQNRRVKHKKEG
Xla_Gsx2        QAXQNGKRMRTAFSSTQLLELEREFSSNMYLSRLRRIEIATYLSLSEKQVKIWFQNRRVKHKKEN
Mmu_Gsx2        QVXPNGKRMRTAFTSTQLLELEREFSSNMYLSRLRRIEIATYLNLSEKQVKIWFQNRRVKHKKEG
Pex_Gsx         XXXXXSKRIRTAFDSRQLLDLEREFGANMYLSRLRRIEIANSLSLTEKQVKIWFQNSRXXXXXXX
Nco_Gsx1        SHAATKRRMRTAFTSRQLLELERQFGANMYLSRLRRIEIAACLQLSEKQIKIWFQNRRVKYKKEX
Nco_Gsx2        DKPPGGKRARTSFTSEQLIELERHFRSNEYLGRTPRVEMALALNLSERQIKIWFQNRRMKQKKER
Plo_Gsx1        HHHVTRRRMRTAFTSRQLLELERQFNANMYLSRLRRIEIAACLQLSEKQIKIWFQNRRVKYKKEX
Plo_Gsx2        TDGAAAKRRRTIFSSEQLIELERHFRTNEYLGRTPRIEMALALNLSEQQIKIWFQNRRMKQKKER
Pdu_Xlox        DFTDENKRTRTAYTRSQLLELEKEFHFNKYISRPRRIELASMLSLTERHIKIWFQNRRMKWKKDE
Bfl_Xlox        AVEDENKRTRTAYTRGQLLELEKEFHFNKYISRPRRIELAAMLNLTERHIKIWFQNRRMKWKKEQ
Sko_Xlox        QDLDENKRTRTAYTRSQLLELEKEFHFNKYISRPRRIELAAMLNLTERHIKIWFQNRRMKYKKEE
Spu_Xlox        ADFDENKRTRTAYTRGQLLELEKEFHFNKYISRPRRIELAAMLNLTERHIKIWFQNRRMKWKKEE
Pma_Xlox        PIEDDNKRTRTAYTRGQLLELEKEFHFNKYISRPRRIELAAMLNLTERHIKIWFQNRRMKWKKDE
Xla_Xlox        MEQEENKRTRTAYTRAQLLELEKEFLFNKYISRPRRVELAVMLNLTERHIKIWFQNRRMKWKKEE
Mmu_Xlox        AEPEENKRTRTAYTRAQLLELEKEFLFNKYISRPRRVELAVMLNLTERHIKIWFQNRRMKWKKEE
Cte_Xlox        QTFSENKRTRTAYTRAQLLELEKEFHFNRYITRPRRVELAAHLNLTEQHIKIWFQNRRMKWKKDV
Hro_Xlox        HILDDNKRTRTAYSRSQLLELEKEFHFDKYISRPRRVELASSLNLTERHIKIWFQNRRMKWKKME
Nco_Xlox        RMFDENKRTRTAYTRAQLLELEKEFHFDKYISRARRLELANLLRLTERHIKIWFQNRRMKWKKYE
Plo_Xlox        RTFDENKRTRTAYTRAQLLELEKEFHYDKYISRARRLEMAKVLRLTERHIKIWFQNRRMKWKKYE
Cte_Cdx         GKTRTKDKYRIVYSEYQKVELEKEYLYSKYITIQRKAELSRSIGLSERQVKIWFQNRRAKERKQK
Pdu_Cdx         XKTRTKDKYRVVYTDHQRLELEKEFHYSRYITIRRKAELAQTLNLSERQVKIWFQNRRAKERKQN
Avi_Cdx         XKTRTKDKYRVVYTDHQRLELEKEFHYSRYITIRRKAELAQNLNLSERQVKIWFQNRRAKERKQN
Bfl_Cdx         GKTRTKDKYRVVYSDHQRLELEKEFYSNKYITIKRKVQLANELGLSERQVKIWFQNRRAKQRKMA
Sko_Cdx         GKTRTKDKYRVVYTDHQRLELEKEFHYSRYITIRRKAELAHALGLSERQVKIWFQNRRAKERKQN
Spu_Cdx         GKTRTKDKYRVVYTDHQRLELEKEFHYSRYITIRRKSELALALGLSERQVKIWFQNRRAKERKMA
Nco_Cdx1        GKTRTRDKYRTVYTEPQRVELEAEFQRTTYISAQRKADIASMVGLSERQVKIWFQNRRAKDRRHD
Nco_Cdx2        GKTRTKDKYRIVYTELQKVELEKEFLFNQYITIQRKAELAGTIGLSDRQVKIWFQNRRAKERKHK
Plo_Cdx2        GKTRTKDKYRIVYTELQKVELEKEFLYNQYITIQRKAELAGHIGLSDRQVKIWFQNRRAKERKHK
Plo_Cdx1        GKTRTKDKYRTVYTDKQRLELEAEYIRATYVSMNRKLNLSCTTGLSERQVKIWFQNRRAKDRRRQ
Pvu_Cdx         GKTRTKDKYRVAYTDHQRLELEKEFHYSRYITIRRKAEIAAQLALSERQVKIWFQNRRAKERKQN
Tca_Cdx         GKTRTKDKYRVVYTDHQRVELEKEFYYSRYITIRRKAELANSLGLSERQVKIWFQNRRAKERKQV
Mmu_Cdx1        GKTRTKDKSRVVYTDHQRLELEKEFHYSRYITIRRKSELAANLGLTERQVKIWFQNRRAKERKVN
Mmu_Cdx2        VKTRTKDKYRVVYTDHQRLELEKEFHFSRYITIRRKSELAATLGLSERQVKIWFQNRRAKERKIK
Mmu_Cdx4        GKTRTKEKYRVVYTDHQRLELEKEFHCNRYITIRRKSELAVNLGLSERQVKIWFQNRRAKERKMI
Xla_Cdx1        TKTRTKDKYRVVYTDQQRLELEKEFHYSRYITIRRKAELAVNLGLSERQVKIWFQNRRAKERKIN
Xla_Cdx2        SKTRTKDKYRVVYTDQQRLELEKEFHYSRYITIRRKAELAVGLGLSERQVKIWFQNRRAKERKIN
Xla_Cdx4        GKTRTKEKYRVVYTDHQRLELEKEFHYSRYITIRRKTELAANLRLSERQVKIWFQNRRAKERKLF
                       :    :   * ::**  :    *:    : :::    *:::::****** *
```
